# Supplementary material for: A systematic strategy for identifying causal single nucleotide polymorphisms and their target genes on Juvenile arthritis risk haplotypes
Source: BMC Med Genomics. 2024 Jul 12;17:185. doi: 10.1186/s12920-024-01954-z (PMC11241977; doi:10.1186/s12920-024-01954-z)
Supplement: Supplementary file 1 — Supplementary Material 1 [file 12920_2024_1954_MOESM1_ESM.docx]

**Supplementary Figure 1** - Western blot showing induction of the Cas9 protein by doxycycline (top image) in K562 cells transfected with the dCas9-KRAB construct after treated with 1 ug/ml of doxycycline for 48h.  The bottom image show the a blot for beta actin to control for efficiency of protein loading. The image was acquired using the FluorChem E imagining system and ProteinSimple version:3.1.0 software.


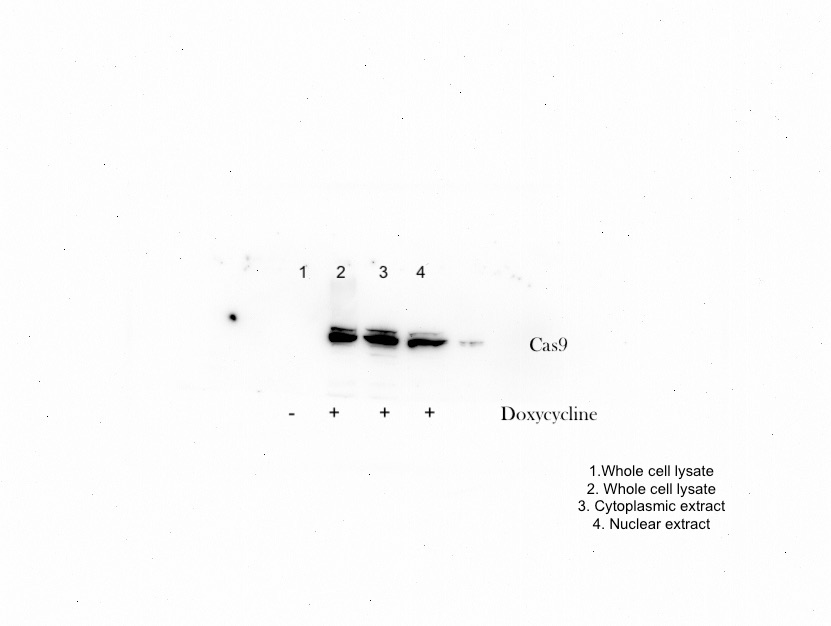


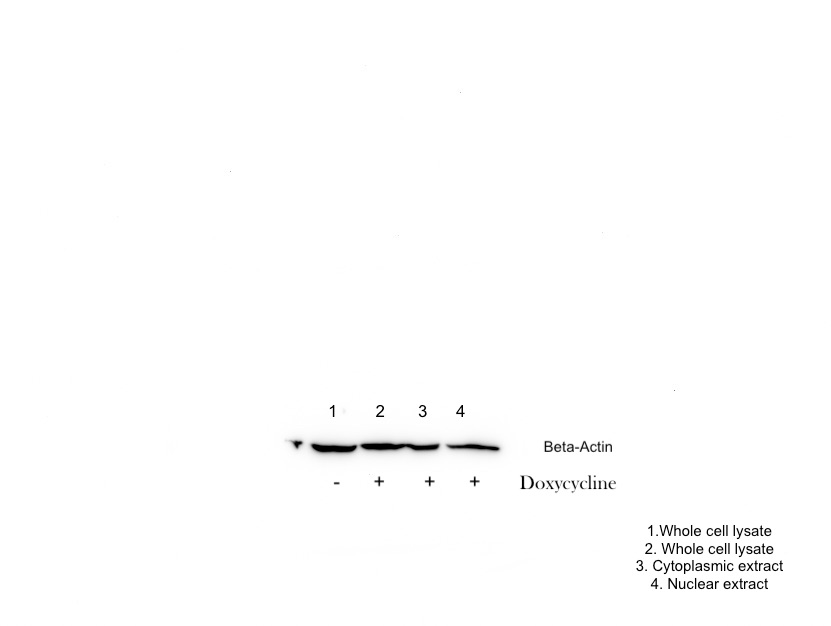


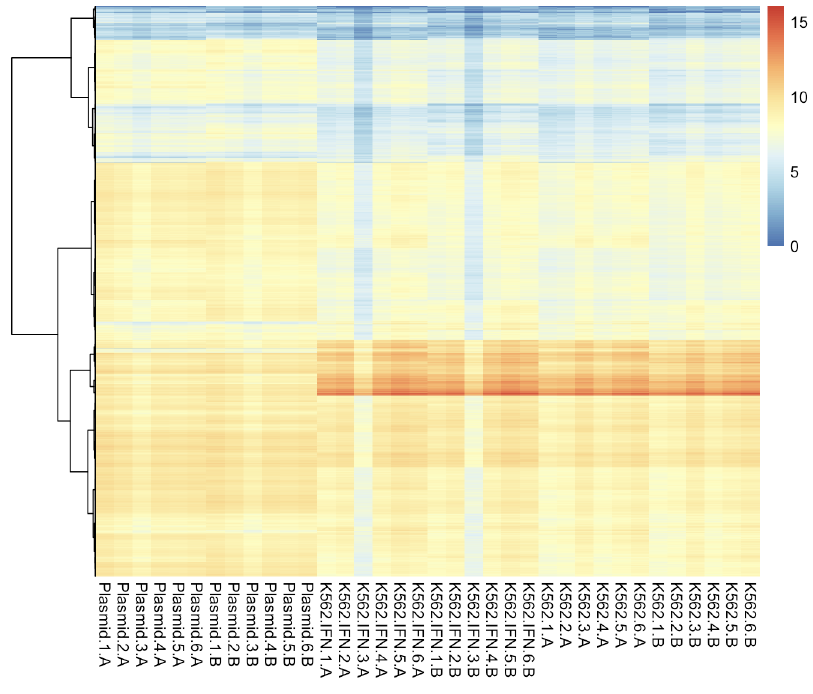


**Supplementary Figure 2** – Heat map showing gene expression levels for each replicate experiment, comparing empty plasmid and reporter vectors (with and without IFNγ stimulation).
